# The Effectiveness of Depth Data in Liveness Face Authentication Using 3D Sensor Cameras †

**DOI:** 10.3390/s19081928

**Published:** 2019-04-24

**Authors:** Ghazel Albakri, Sharifa Alghowinem

**Affiliations:** College of Computer and Information Science, Prince Sultan University, Riyadh 11586, Saudi Arabia

**Keywords:** face authentication, liveness assurance, biometric technology, 3D face authentication, anti-spoofing techniques

## Abstract

Even though biometric technology increases the security of systems that use it, they are prone to spoof attacks where attempts of fraudulent biometrics are used. To overcome these risks, techniques on detecting liveness of the biometric measure are employed. For example, in systems that utilise face authentication as biometrics, a liveness is assured using an estimation of blood flow, or analysis of quality of the face image. Liveness assurance of the face using real depth technique is rarely used in biometric devices and in the literature, even with the availability of depth datasets. Therefore, this technique of employing 3D cameras for liveness of face authentication is underexplored for its vulnerabilities to spoofing attacks. This research reviews the literature on this aspect and then evaluates the liveness detection to suggest solutions that account for the weaknesses found in detecting spoofing attacks. We conduct a proof-of-concept study to assess the liveness detection of 3D cameras in three devices, where the results show that having more flexibility resulted in achieving a higher rate in detecting spoofing attacks. Nonetheless, it was found that selecting a wide depth range of the 3D camera is important for anti-spoofing security recognition systems such as surveillance cameras used in airports. Therefore, to utilise the depth information and implement techniques that detect faces regardless of the distance, a 3D camera with long maximum depth range (e.g., 20 m) and high resolution stereo cameras could be selected, which can have a positive impact on accuracy.

## 1. Introduction

The metrics that assess measurements of body identifiers of a person to distinguish them from others are called biometrics. These biometrics are unique for each individual and are therefore, used as a technology to secure systems for authenticating (identification) purposes. Common examples of biometrics are fingerprint, iris pattern, voice, face and signature. Since technology has become a critical part of our life, securing it with biometrics-based authentication systems that are also equipped with anti-spoofing techniques is essential. Presently, spoof attacks are rapidly expanding. Mock-ups of real human fingerprints are created using silicone and are later used in deceiving and accessing fingerprint-based devices. Moreover, fake masks of different material are being used to access face recognition systems. Such attacks are at hand for many people due to the fact that the materials used in such attacks are affordable and within reach of everyone. For this reason, biometrics devices must have robust security approaches. Liveness assurance methods aim at protecting biometrics devices from such spoof attacks. When a biometric is scanned, various approaches are implemented to determine the liveness of this biometric. Measuring heartbeats, estimating 2D and 3D face proportions, evaluating the quality of scanned faces and detecting heat radiating from scanned biometrics are all effective anti-spoofing techniques.

Liveness assurance of face using real depth information is not commonly used in biometrics technology and the literature. Datasets consisting of different video and photo spoof attacks exist for research evaluation. All publicly available datasets such as CASIA Face Anti-Spoofing DB, NUAA Photograph Impostor DB and PRINT-ATTACK DB are designed for 2D spoofing prevention and therefore the images in these datasets are 2D images, and no depth data is included in these datasets. While 3DMAD, IIIT-D and BUAA Lock3DFace datasets contain real depth data, papers found discussing these datasets did not use depth to analyse them, but instead used different approaches such as dynamic texture analysis and remote photoplethysmography (as discussed later).

Moreover, the technique of using real depth data in 3D liveness detection is not commonly implemented in face recognition devices and systems. Face recognition systems use other approaches such as 3D estimations through software solutions, colour texture analysis and image quality assessment to detect spoof attacks. It is worth noting that the technique of 3D liveness detection using real depth is rarely evaluated and therefore this research will focus on evaluating this technique by reviewing face liveness detection techniques and highlights their weaknesses.

Following our proposed method in [1], we aim to evaluate the real depth anti-spoofing methods and analyse its reliability for liveness detection. This is done through a literature review of current face authentication, where liveness detection is investigated. In this work, beside reviewing the literature of this aspect, we conducted proof-of-concept experimental tests to assess the depth information in the face authentication technique and evaluated its capability in distinguishing fake biometrics.

## 2. Background

Subject dependent and subject independent are two methods used in statistics for samples collection. In the user independent samples, the values in the sample have no relation to the other values in the same sample; the researchers compare results of different groups to each other. For example, a group of individuals with blood type AB+ and a group of individuals with blood type O- both participate in a study that observes the effect of a medication on both groups’ blood pressure, the study compares the results of the first group with the results of the second group. These two groups are not related to each other and therefore are considered independent samples.

On the other hand, in user dependent samples a set of items is paired with the samples of the same subject to measure the accuracy and compare the values. Values in the user dependent samples affect other values in the same sample. For example, a group of researchers are studying the effect of emotion triggering videos on depressed people. To do so effectively, the researchers will record the details of the depressed individuals before watching the videos and then compare it with the details of the same depressed individual after watching the videos. In this case, the data is user dependent because researchers are comparing a sample of data with the same sample of data in different situations.

Biometrics technology follows user-dependent sampling process. To validate scanned biometrics, the user’s scanned image is compared with the same user’s biometrics stored in the system. For example, if the user logs into the system using their fingerprint, the systems measures the similarity between the scanned fingerprint and the fingerprint registered for the user that is stored in the system’s database. Validating biometrics is accomplished using the universal background model (UBM). UBM is a commonly used with Gaussian mixture model (GMM) that is used as a discriminative approach, where the authenticity of the sample is determined by running a comparison between user dependent characteristics and a sample of all other users. As proven by other researchers, UBM is helpful in speaker-independent models, and is trained to use big data collected from diverse subjects [2,3,4].

**Figure 1:**
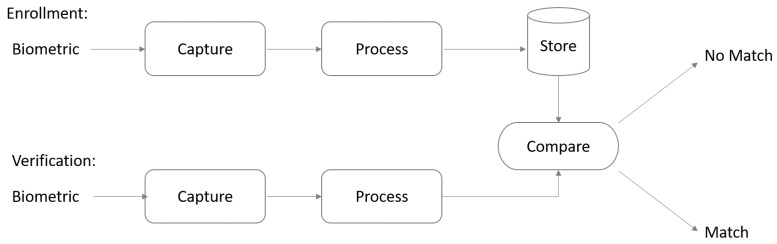
Biometric enrolment and verification process.

Figure 1 shows the enrolment and verification process, where the first step in registering biometrics is to capture the user’s biometrics. The scanned biometrics are then processed by the system and its distinctive characteristics are recognised and registered in the system’s database to be used in the comparison process. The system follows certain steps to verify the scanned user’s face during the log in process. Unique features for the scanned biometrics are recognised and compared with the biometrics stored in the system’s database. The system successfully logs the user in once the scanned biometrics is matched with the stored biometrics.

Different measurements could be used for biometric systems performance evaluation. For example, the receiver operating characteristic (ROC) curve examines a model accuracy and ability to differentiate between authentication cases (i.e., acceptance and rejection). The ROC curve plots true acceptance rate versus false rejection rate [5]. Through ROC, all possible threshold values could be analysed to set the level of a system security [6].

False acceptance rate (FAR) referrs to the state when a system incorrectly recognises an unauthorised user. It is the rate of allowing access to an unauthorised user and labelling them as genuine and authorised [7]. It is calculated by dividing the number of false acceptances by the total number of log in attempts.

(1) $$FAR=\frac{\sum \mathrm{Number}\;\mathrm{of}\;\mathrm{False}\;\mathrm{Acceptance}}{\mathrm{Number}\;\mathrm{of}\;\mathrm{Attempts}}$$

False rejection rate (FRR) is the state of rejecting an authorised user. When the user is real and should be authorised but the system considers the user as false and denies access [7]. It is calculated by dividing the number of false rejections by the total number of log in attempts.

(2) $$FRR=\frac{\sum \mathrm{Number}\;\mathrm{of}\;\mathrm{False}\;\mathrm{Rejection}}{\mathrm{Number}\;\mathrm{of}\;\mathrm{Attempts}}.$$

### Liveness Assurance/Detection

Biometric systems are prone to spoofing attacks (e.g., fake silicone fingerprint), which is the reason that several anti-spoofing methods are implemented to recognise and prevent such attacks. Such anti-spoofing techniques aim at assuring that the scanned biometric is from a live person (e.g., detecting pulse). Anti-spoofing techniques are varied between biometric type. Facial spoof cases such as silicone masks, 2D printed face images, and face images displayed on screens can all be detected using one of the following techniques.

Estimation of pulse has been used in liveness detection of faces compared to still faces. Heart rate could be estimated remotely from face videos using the photoplethysmography (PPG) technique [8,9]. PPG measures heart pulse by detecting the light absorbed by the haemoglobins, which influences the skin colour. The cardiac pulse adjusts the haemoglobins count within a local part of skin. Therefore, it has been used in face liveness detection.

Another technique for face liveness assurance is by assessing image quality of the face, which is a common approach for fraudulent face detection. Galbally et al. [10] stated that a face image that is captured using a mobile is probably over- or under-exposed. Therefore, such images have specific characteristics that distinguish them from real images.

Recently, analysing the colour texture of the chroma channels has been investigated for recognising authentic face biometrics. This is due to the fact that real faces colour spectrum differs from face images and masks colour spectrum. While genuine faces have a wide gamut rang, fake faces contain local variety of colours. According to Boulkenafet et al. [11] colour texture analysis is the process of extracting LBP features from the face images and analysing its micro-texture representation using a support vector machines (SVM) classifier.

Light reflection of face image was also used in identification of spoof attacks. Mhou et al. [12] proposed a method that employs night vision infrared cameras in detecting fraudulent biometrics. Along with the infrared cameras, LPB algorithms, blur detection, median blur filter and Gabor filters were used in the identification process. The face biometric captured by the camera is analysed through the proposed algorithm to detect the liveness of the face. After capturing the image it goes through a Gabor filter, a filter that is commonly used in edge detection, obtaining features and analysing text. The next step in the presented system is detecting image blurriness using the Laplacian variance, this is achieved by calculating the standard deviation and measuring its square. Later the image texture and colour is measured to determine its authenticity.

To avoid spoofing attacks, an estimation of the 3D depth of the face was investigated for liveness assurance. Kittler et al. [13] specified that sensing technologies could be utilised to reconstruct a 3D measure of a face by projecting a structured illumination pattern. Additionally, a 3D facial acquisition mostly depends on time-of-flight; the time needed for the projected illumination to reflect from the scanned face and on triangulation; determining the location of certain points on the scanned face images by measuring the distance between points of angles.

## 3. Face Recognition Datasets

We will briefly introduce existing datasets used in face recognition and liveness detection research. These datasets are used in papers discussed in related work section. A summary of the reviewed datasets discussed in this section can be shown in Table 1.

- ZJU eyeblink dataset.
  This dataset is a video database that contains 80 video clips in AVI format of 20 candidates. Each subject had four clips in this dataset; the first clip was a clip for front view of the candidates without glasses, the second clip was a clip for front view of the candidates wearing rim glasses, the third was a clip for front view of the candidates wearing black glasses, the fourth and last clip was a clip of the candidates looking upwards without any glasses. As a liveness indicator, all candidates performed spontaneous eye blinking in normal speed in all four clips. Each video clip lasts about five seconds and the number of blinks per clip varies from one to six times. Data were captured indoor without illumination control with a total number of 255 blinks.
- Print-attack replay dataset.
  This dataset consists of 200 video clips of 50 candidates under different lighting conditions and 200 real access videos of the same candidates. The data is split into four subcategories. Enrolment data, training data, development data, and test data to validate spoof detection. Each subcategory contains different candidates. High resolution images of candidates are used to generate photo attacks.
- In-house dataset for Sant Longowal Institute of Engineering and Technology (SLIET).
  This dataset is developed by Manminder Singh and A.S. Arora with eye blinking, lip and chin movements as liveness indicator. It contains 65 video clips of students in the institute. Different lighting conditions are applied when capturing video clips and photos of the students to generate photo attacks. The dataset contains two categories: the first includes videos of real candidates and the second category contains photos of the candidates held by the attacker.
- Replay-attack database.
  This dataset contains 1300 video clips of photo and video attacks of 50 candidates. Photos and video clips are captured under different lighting conditions. The data is split into four subcategories. Enrolment data, training data, development data, and test data to validate spoof detection. Each subcategory contains different candidates.
- NUAA dataset.
  A public face spoof attack that contains 15 candidates with recorded bona fide face videos. Candidates were recorded on three sessions per candidate, each session consists of 500 samples. High resolution photos of candidates are included in this dataset as well. These photos were printed on A4 paper.
- CASIA face-anti-spoofing database.
  This dataset consists of 50 candidates with video recording of each candidate with genuine and fake faces. Genuine faces were captured from real users while the fake faces were captured from high resolution recordings. Three main spoof attacks were designed; by each candidate; warped photo attacks, photo masks and video attacks. Facial motions and eye blinking were performed to produce liveness illusion. Data were collected under three different resolutions: low, normal, and high.
- The 3D mask attack database (3DMAD).
  This dataset is a public 3D mask database. It contains 76,500 frames of 17 candidates. Photos are captured in a way that gives depth and colour. Each frame entails the depth, RGB and eye position of the candidate. The data is collected in three different sessions for all candidates and videos are recorded under controlled conditions. Session one and two were genuine access attempts by candidates, while the third session was a recording of an attacker with 3D mask attacks. Moreover, the dataset includes face photos used to generate masks and paper-cut masks.
- Antispoofing dataset created by Patrick P. K. Chan.
  This dataset consists of 50 candidates; 42 male and eight female with ages from 18 to 21. There were two images taken for each candidate; one with a flash and another without. The images were taken seconds apart with different lighting conditions and various distance settings. Five types of 2D spoof attacks are included in this dataset; paper photo attack, iPad photo attack, video attack, 2D mask attack and curved mask attack. A hot object was placed on the top of the spoof papers to increase its temperature and create a liveness illusion.
- MSU mobile face spoofing database (MSU MFSD).
  This dataset contains 280 videos of 35 candidates. This dataset is divided to two categories: training and testing. Spoof attacks consists of photo and video attacks consisting of printed attacks, replay attacks generated with a mobile phone and a tablet.
- Replay-mobile database.
  This dataset consists of 1030 videos of 40 candidates. Spoof attacks contain print photo attack, tablet photo attack and mobile video attack. Three subcategories are included in this dataset: training, development and testing.
- IIIT-D.
  This dataset is a public dataset that consists of 4603 RGB-D images of 106 candidates, the images were collected under normal lighting condition with few pose and expression deviations.
- BUAA Lock3DFace.
  This dataset is a public dataset that includes 5711 RGB-D video sequences for 509 candidates. The videos were recorded in different poses, expressions, constrictions and time.
- Dataset created by Jiyun Cui.
  Jiyun Cui et al. [14] created a dataset using RealSense II. Their dataset includes about 845K RGB-D images of 747 candidates with various poses and a few lighting conditions. The dataset is randomly divided into training and testing candidates.
- Silicone mask attack database (SMAD).
  This dataset is presented in [15]. It consists of 27,897 frames of video spoof attacks of vivid silicone masks with holes for the eyes and mouth. A few masks have hair, moustaches, and beards to create a genuine illusion for life-like impressions. This database comprises 130 videos, 65 genuine samples, and 65 masked samples. Image samples of real faces and fake silicon faces are included in this dataset as well.
- Oulu-NPU database.
  This database contains 4950 bona fide and artefact face videos of 55 candidates. Front cameras of six mobile devices (Samsung Galaxy S6 edge, HTC Desire EYE, MEIZU X5, ASUS Zenfone Selfie, Sony XPERIA C5 Ultra Dual, and OPPO N3) are used to record the bona fide samples. These samples were captured under different lighting conditions. While artefact species were captured using various types of PAIs, including two different kinds of printers and a display screen.
- Yale-Recaptured Database
  This database is a recaptured and extended dataset of Yale Face Database B, which consists of 16,128 frames of 28 candidates. The photos taken of candidates includes 9 poses and 64 lighting condition per candidates.
- MS-Face Database
  This dataset is a multispectral face artefact database which includes images collected from 21 candidates under different imaging settings. For the bona fide dataset, each candidate was captured in five various conditions. The spoof attacks were designed by presenting three printed images of the best quality in the visible spectrum under three different illumination settings: natural, ambient lighting, and two spotlights.
- Florence 2D/3D face dataset.
  This dataset is created with the purpose of supporting and is therefore designed to replicate genuine surveillance conditions. The dataset contains 53 candidates with high-resolution 3D scans, video clips with different resolutions and zoom levels. Each candidate was recorded in three different conditions: video clips in an organised setting, indoor setting with minimal constraints, and outdoor setting without constraints. This dataset was not designed for spoofing purposes and therefore no liveness indicators nor spoof attacks were included.

Several papers used the above datasets. For 3DMAD, IIIT-D and BUAA Lock3DFace depth was recorded, however, the papers that used these datasets have different approaches such as dynamic texture analysis and remote photoplethysmography. Additionally, a number of papers [15,16,17] have used 3DMAD dataset, nevertheless, these papers have not used depth as a liveness detection method in their studies. This is our main motive in conducting this research paper, we aimed to investigate the reason behind the lack of use of this approach. Evaluating the effectiveness of using real depth data as a liveness detection was an essential step to answer this question.

## 4. Related Work

We review in details all the research work that investigated liveness detection from face biometrics. We explore their used authentication techniques and discover their weaknesses. Table 2 summarises the related work discussed here.

In their research, Wasnik et al. [19] tested the security of smart-phone based facial authentication methods. A new approach was presented for spoofing attacks detection on iPhone 6s, which has been achieved through investigating the sensor raw features of the smart-phone camera. Sensors are usually in the form of colour filter arrays that has dedicated cells for blue, green and red spectrum data. In their proposed method, they captured the data onto the sensor where they divide the image to red, blue and green channels to analyse its features at the sensor level data. This is achieved using a simple block-wise energy. The proposed approach eliminated the need for a specially learnt classifier.

Pravallika and Prasad [20] applied SVM classification to iris, face and palm print using image quality assessment to detect fake biometrics. In their study, the authors used image fusion, the process of combining biometric traits such as palm print and face, into a single image and checked for authenticity. The image was analysed afterwards and patterns are recognised using SVM. The results in their research indicated that SVM classifier was efficient and accurate in recognising real and mock biometrics.

Image quality assessment (IQA)-based method has been introduced by Patil and Dhole [21] to detect fake multi-biometrics. The research proposed a novel software based method to detect mock fingerprints synthesised from Fevicol and fake face images such as photo printed images, and high and low quality mobile images. The system was able to efficiently assist in detecting fraudulent samples and overcome the lack of generality in liveness detection techniques. This has been achieved using measurements acquired based on pixel information, correlation between images, edge based information and visual quality loss.

In their research, Galbally et al. [10] have also experimented with fraudulent biometrics detection using IQA. Their study has been applied to iris, fingerprint and face biometrics. Gummy fingers were created using silicone, gelatine and play-doh and mock iris samples were obtained from a database of fake biometrics. Moreover, face spoof attacks were implemented using printed hard copies of high quality images, on-screen face images and videos displayed on iPhone screen and high resolution images and videos displayed on iPad screen. Their research aimed to enhance the security of biometrics recognition frameworks and was able to correctly classify over 97% of the samples. The results has shown the high potential of image quality assessment for securing biometric systems against a variety of attacks.

Kim et al. [22] presented an approach to intercept image and video attacks through using a regular charge-coupled device (CCD) or USB cameras. Their proposed approach assumed the background ought to possess information that is important for detecting fake images and biometrics. Their proposed approach showed good results for detecting image and video attacks.

Killioǧlu et al. [18] investigated liveness detection through a pupil tracking system in face recognition systems. In their approach, real-time Haar Cascade classifier was used to extract eye area. The proposed algorithms have been tested and achieved 98% success rate.

Wang et al. [23] suggests a method for face liveness assurance by analysing the 3D structure of the face using SVM classifier, which achieved 100% classification results. However, the method was not able to generalise when using other devices, where the image quality differ from the samples used in training the model. Therefore, liveness detection results were greatly less than expected.

Focusing on 2D liveness detection technique, Yan et al. [24] explored three liveness clues in temporal and spatial domain. The clue of non-rigid motion analysis resulted in an accuracy of 90%, while for the face-background consistency analysis, the accuarcy was 97.50% and for the image banding analysis was 97.50%, which indicates the effectiveness of their method. However, each clue is applied based on the background and surrounding environment and therefore these clues give best results when used independently and cannot be combined and used together.

Kollreider et al. [25] used optical flow of lines and introduced optical flow pattern matching to determine liveness of scanned faces. The suggested approach exploits the image motion features by analysing a captured face image sequence and identifying whether it is real or fake. The approach had precise measurements for anti-spoofing assessment, however, only for sequences generated from horizontal movements. Furthermore, eyeglasses and facial hair affected the flow patterns and caused wrongful localisation.

Spoof biometrics detection based on the behaviour of noises was proposed, Nguyen et al. [26] used noise local variance model on smart-phones. The distribution of noise perform differently between real and fake face images. The statistic behaviour of this distribution of noise had an encouraging performance in liveness detection.

Karthik and Katika [27] also proposed image quality assessment to detect fake biometrics. The tests were performed on a contrast 2D printed photograph by spoof attacking using wrapped and unwrapped images. The results proved it is possible to achieve an equal error rate (EER) of 21.56% and false acceptance rate (FAR) of 8.57%. However, this approach has not been tested on other spoofing attacks such as fake face masks and face images displayed on screens.

Chan et al. [39] used a flash light on a face capturing system to improve the liveness assurance in 2D manner. This allows for analysing face image texture and structure in response to light reflection. They attempted to attack the system using printed face images, video attacks, photographs displayed on iPad, 2D masks and curved mask. Their method outperformed existing anti-spoofing techniques, confirming the enhancement effect of using flash in face liveness detection. However, flash light radiance can be disturbing to the users’ eyes, which might affect the acceptance of such technology.

Mhou et al. [12] proposed a method that also utilises the light reflection patterns for fake biometrics detection. The implemented system uses Laplacian blur detection, Gabor filters, colour moments and local binary patterns as features. The reflection of light is measured on different face values to identify real images. Even though the results illustrated the effectiveness of the method, the light intensity affects the classification. Moreover, detecting fake images was somewhat more efficient when analysing images captured using infrared camera compared to images obtained using normal cameras.

In an approach to detect fake facial biometrics, Lakshminarayana et al. [28] investigated using spatio-temporal mapping as an anti-spoofing method. Their proposed method analysed distinct characteristics that indicate liveness of the face image using a convolutional neural network (CNN). They created a system that calculates attributes which define the liveness of an individual. The results affirmed that the proposed model surpassed other methods and exploited few key features of liveness which reduced model complexity. On the other hand, the method requires higher time to compute.

Tirunagari et al. [29] modified dynamic mode decomposition (DMD) for anti-spoofing, where DMD is able to recognise liveness features such as eyes blinking, lips movement and dynamic face features. The approach uses local binary pattern histograms and SVM classifier. The proposed approach captured the unique features produced by print and replay attacks compared to live videos.

Using deep learning architecture, representations of deep iris, face and fingerprint are used to build image-based spoof detection systems Menotti et al. [30]. Two deep learning methods were analysed, which are architecture optimisation and training filter. The approach was successful for fingerprint verification, however, further improvement is needed for iris and face liveness detection.

Arashloo et al. [31] detected spoof biometrics using a dynamic texture descriptor, where liveness is detected through a discriminative representation of the dynamic multiscale binarised image. Their method enhanced the liveness detection performance. However, the error rate increased for printed face images, where eyes positions were cut. This kind of spoofing aims at simulating the effect of eye blinks.

Boulkenafet et al. [32] investigated the effectiveness of detecting fake biometrics by analysing the colour texture of the scanned face. Utilising the face colour texture improved the robustness of the descriptors, which resulted in achieving reliable performance. However, the method did not include face normalisation or face bounding box, which are essential factors in intra-database tests.

Aziz et al. [33] used the physical properties of photographs, where images were captured at different angles using Stokes parameters; values of the polarisation state of electromagnetic radiation. The obtained values were calculated using the Stokes degree of linear polarisation (SDOLP) and analysis was performed to evaluate the colour of the skin and figure its relation in detecting fake face images. However, the proposed method could not generalise for dark skin.

Di Wen et al. [34] analysed four types of image misrepresented features for anti-spoofing (specular reflection, blurriness, colour moments, and colour diversity). Two types of attacks were investigated: printed photo, and replayed video with different mobile devices. Even though the results exceeded the performance of other anti-spoofing approaches, the approach was tested on small sample where the illumination of surroundings were not considered.

Raghavendra et al. [35] used the spectral signature of the scanned face image in characterising the reflectance properties. The results showed outstanding performance for the suggested approach. However, the face’s spectral features such as cheeks and eye area were not considered, which plays a vital role in detecting liveness of the face.

Li et al. [9] used pulse estimation to detect fake facial biometrics in videos. The experiment implemented three different types of attacks: printed images, 3D masks and videos. The approach measured heartbeats using photoplethysmography (PPG) technique. Facial colour changes were analysed, that indicate the existence of cardiovascular heartbeats. These characteristics are capable of recognising spoof face biometrics. However, when textural information was analysed miss-classification errors occurred with half of the attacks.

Benlamoudi et al. [36] utilised pattern recognition process for face anti-spoofing. Fisher score was used to reduce the dimensionality of the total of histogram bins of the extracted features. Then, a radial basis function kernel SVM classifiers was used for the recognition task. Even though the proposed method was effective for normal and low quality images, it was not effective in high quality face images.

Mohan et al. [37] combined histogram of oriented gradient-local phase quantisation (HOG-LPQ) with fuzzy logic-based SVM Fuz-SVM classifier. The proposed system was efficient in determining fake and real biometrics. However, generalising the method using different dataset was not investigated for further enhancements.

Galbally et al. [38] tested the robustness of the 3D liveness detection technique used in ArtecID 3D Face LogOn and compared the performance of 2.5D and 3D face recognition systems. Nevertheless, anti-spoofing technique as used in the 3D commercial face recognition system is not known. Additionally, they only used 3D printed masks as a spoof attack and the objective of their work is not to develop a new face recognition system but to evaluate the robustness of existing face recognition systems.

Chan et al. [39] investigated using flash to enhance the detection of 2D spoof attacks. The proposed model applies SVM and the Gaussian kernel implemented by libSVM as classifier in their experiments. Five different types of 2D spoofing attacks were performed: (1) A4 paper photo attack, (2) iPad photo attack, (3) video attack, (4) mask attack, and (5) curved mask attack. Using flash enhancement, the spoof attack detection and the proposed method had satisfying results. However, illuminating flash might cause user discomfort which may limit the application of the proposed method. Additionally, using flash has been tested against 2D face attacks only, its effectiveness has not been tested on 3D masks and face attacks. Singh and Arora [40] proposed a novel face detection algorithm in their paper. The proposed algorithm records eyeblink, lip movement and chin movement as liveness indicators and analyses changes in the sequence of frames to detect motion, but 3D data is not included. Moreover, a static background frame is compared with the current frame of a video sequence on a pixel by pixel basis to detect motion. Three datasets have been used to validate and test the proposed algorithm, ZJU Eyeblink dataset, print-attack replay dataset which includes video and photo attacks and an in-house dataset. The proposed method in conjunction with multiple liveness indicators improves the security of face recognition system. Nevertheless, the proposed method does not use real depth data, this area still remains undiscovered.

In [41], the authors presented a simple liveness detection method to improve 2D face recognition techniques and increase their robustness against spoof attacks. Through experiments the authors uncovered that using additional sources of light such as flash can be effective in detecting spoof attacks and can be used as an authentication framework. The authors aimed to investigate the effectiveness of computing 3D features without the need to do a full 3D reconstruction. A dataset was created with 17 subjects in different environments and lighting conditions. The focus of their work is to study liveness detection by exploiting faces’ reflection features. Validation experiments indicated that the proposed approach provides a more reliable facial representation than the existing alternatives.

In [42], the authors proposed an efficient approach for training deep CNN classifiers for face liveness detection. Deep CNN networks were trained using end-to-end learning on databases with limited training samples like face anti-spoofing databases. CASIA-FASD and replay-attack databases were used in their research. According to the authors, the proposed approach proved reliable in both intra-database and cross-database face liveness detection problems. Results revealed that the proposed approach lowers the HTER by 8.28% and 14.14% in cross-database tests. However, this work is classification by 2D image examples only.

Cui et al. [14] proposed an end-to-end learning approach to estimate face depth information from a 2D face image. The proposed approach improves the accuracy of 2D face recognition by utilising the estimated depth information. Results show that the proposed method achieved depth estimation results parallel to the state-of-the-art method and achieved better performance of face recognition.

In [43], the authors proposed using guided scale texture to detect spoof attacks where the scanned image is mapped to the guided scale space before feature extraction and the unnecessary noise components are reduced, while the effective facial edges are preserved. Experiments are done with public MSU MFSD, CASIA FASD, replay-attack and replay-mobile databases. Although the proposed method demonstrated promising results both in intra-database and cross-database testing, sampling and quantisation strategies of the proposed descriptors need to be improved.

Boulkenafet et al. [16] presented a novel software-based approach that use colour texture analysis to detect spoof attacks. In their experiment, the colour local binary patterns (LBP) descriptor was used on CASIA database and replay-attack database to distinguish fraud attacks from real faces. The proposed method outperformed gray-scale counterparts in detecting spoofing attacks.

Shao et al. [15] proposed deep dynamic texture; a new feature to detect 3D mask attacks by evaluating the dynamic information of textures from a CNN’s convolutional layer. The proposed method is validated on 3DMAD dataset and the silicone mask attack database (SMAD), 3D mask attacks are available on these datasets however real depth data is not available. The proposed algorithm achieved excellent results which is an indication of the effectiveness of the 3D mask anti-spoofing method.

Alotaibi and Mahmood [44] proposed applying nonlinear diffusion and using specialised deep convolution neural network to extract high-level features of input image and use the obtained information in detecting fake face attacks. In their work the authors used images and videos available in NUAA dataset and replay-attack dataset. Results obtained show that the proposed method achieved an accuracy of 99% on NUAA dataset.

In [17], the authors discussed lessons learned about spoofing and anti-spoofing in face biometrics, common issues and future work have been discussed as well. The replay-attack database is used as a baseline to analyse the performance of the face baseline system. However, the authors did not discuss 3D anti-spoofing techniques. Overall, results demonstrated that most of the biometrics modalities are prone to spoofing attacks with different degrees.

A recent investigation of specific characteristics of display platforms was presented in [45]. The study analysed the image quality and characteristics using several face images and face parts and their colouring profiles to detect the display spoofing attack. By utilising a probabilistic neural network (PNN), the study reached 99% success rate in spoof detection, which is considered state-of-the-art.

Despite the effectiveness of all the above proposed methods, most of these methods did not use biometric hardware and software together for spoof detection from depth information. Additionally, the reviewed researches do not focus on implementing real depth data as a liveness assurance method, instead they focus on implementing new techniques and enhancing existing classifiers and spoof detection methods such as IQA and colour texture analysis from 2D images. Therefore, this work investigates, through a proof-of-concept study, the performance of using real depth data as an anti-spoofing methods. We also explore the strength and weaknesses of using this technique compared to other liveness recognition methods.

## 5. Proof-Of-Concept Study

**Figure 2:**
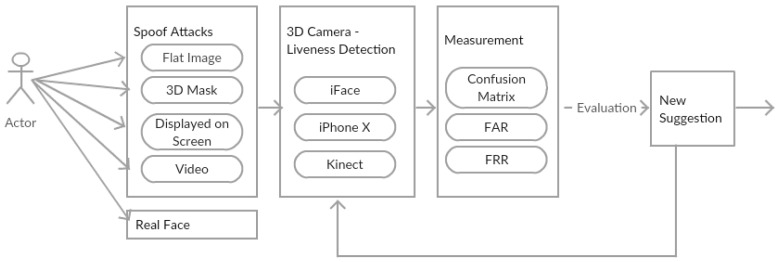
Flowchart of the process.

The proof-of-concept study investigates the effectiveness of anti-spoofing techniques of face biometrics that utilise real depth data in terms of strength and weakness. This is done through comparing three devices that have 3D sensor camera face authentication results when spoofing cases are presented. The Flowchart Figure 2 illustrates the general overview of the proof-of-concept study.

### 5.1. Method

As discussed for any biometric system, the first part of the experiment is to enrol the user on all devices. Only one user is enrolled as a proof-of-concept, and to ensure comparable results between the devices that do not allow more than one user to register. Following the enrolment process the user will attempt to access all three devices, spoof attacks will be performed and also real face to test the devices’ robustness. Every reaction and result is recorded for measurement, we used the confusion matrix, the false acceptance rate (FAR) and the false rejection rate (FRR) to measure the system’s effectiveness and accuracy in detecting fraud attacks. The outcome is then evaluated and based on the results a new suggestion is presented. The new suggestion will be tested against the devices for validation purposes.

A confusion matrix can be defined as a table used to characterise the performance of a classifier on a set of data. The matrix table consists of columns and rows, the columns represent positive and negative predicted classes while the rows present positive and negative actual classes. The intersection between these columns and rows produces: true positives (TP), true negatives (TN), false positives (FP) and false negatives (FN). From the confusion matrix, several measures could be calculated such as accuracy, precision, sensitivity, etc. Beside FAR and FRR described in Equations (1) and (2), accuracy (ACC) measure, which is also used in this research, is calculated as the sum of true positives and true negative, which is then divided by the number of samples in all classes.

(3) $$ACC=\frac{\sum \mathrm{True}\;\mathrm{positive}+\sum \mathrm{True}\;\mathrm{negative}}{\sum \mathrm{Total}\;\mathrm{population}}$$

There are various methods to acquire and enrol 3D face biometrics, one of which is BioID. BioID is a mobile application that applies anti-spoofing algorithms. BioID detects fake facial scans using motion-based anti-spoofing methods. Nonetheless, 3D cameras are not used in this application. All of which does not support our research scope. On another note, 3D cameras are used as face authentication and fraud detection mediums in three different devices. iFace 800 (manufactured by ZKTeco, Dongguan, China),iPhone X (using Face ID) and Microsoft Kinect. The effectiveness of these devices in detecting spoof attacks are explored and tested.

Five categories of spoofing test cases are applied on these devices, which will be explored in this research. Each category implies a different attack method and approach. (see Table 3). Flat printed image casesd experiment with different approaches to access the system using an image of the subject’s face printed on papers of different sizes and qualities. The printed images will be positioned in front of the three face recognition devices. The objective is to test the systems’ reliability in calculating 3D measurements on the printed face images and test the effectiveness of this anti-spoofing technique. In this research we will be using matte and glossy since they are the commonly available types of paper and for their differences in reflecting light, which might affect the recognition results.

In order to consider the deformation and the depth effect that occurs when a printed image is bent, we also include mask attack test-cases. For mask attacks, similar to flat printed images, the mask will be printed on different types of paper material and will be bent and worn as a mask to create the illusion of 3D in a real face.

Flat image on screen and video attack cases were displayed on screens with varied resolutions (see Table 4). These test-cases were designed to examine the effect of screen resolution and material on the scanning and identification process. Moreover, these test-cases were designed to inspect the systems measurements of real depth of the scanned image and videos. For videos, one short video of the user looking directly at the camera with slight head movements and eye blinking to assure liveness were recorded. Our goal was to test whether the video will trick the face recognition devices and to test the effect of size on the recognition process.

Moreover, to test the flexibility of the systems in the three devices, real user face is used with mild alteration. We expected to have a smooth log in process since these alterations are not drastic and they do not affect the features of the user’s face. Besides providing a baseline, these test cases are implemented to test the false positive aspect of the system, we also want to have balanced test cases.

#### Authentication System Using Kinect Sensor

iFace 800 and iPhone X have their own authentication systems, while Kinect needs a customised authentication system. To accomplish our objective of comparing and testing the effectiveness of depth data in spoof attacks detection, we developed a system that uses Kinect Sensor for this task. Our system used a Haar Cascade algorithm to detect and recognise faces that appear on the screen. Open source computer vision library (OpenCV), which is “an open source computer vision and machine learning software library”. The library has more than 2500 optimised algorithms that can be used to detect and recognise faces, identify objects, classify human actions in videos, track camera movements and many other functions [46]. OpenCV is compatible with Kinect Sensor and can be beneficial in face recognition and optimisation.

First time users must first register their face and information. The system benefited from the 3D camera available in the Microsoft Kinect Sensor, the face is scanned and measured using depth camera. When a user stands in front of the camera, the image will be scanned to detect a face, where the system will be performing two tasks in parallel; scan the image and measure the depth.

To measure the depth, five points will be placed across the scanned image. One dot will be placed in the centre of the face image and the other four dots will be placed 30 pixels above, below, on the left and on the right of the centre point. When the measurement for the five points is acquired, the depth values will be compared. The depth values of these points should vary. If two points or more have equal depth values then the face is 2D and will be considered fake. Once a face is detected and depth is measured and verified, the system starts comparing between the detected face and the array of images stored in the library using Haar algorithm.

### 5.2. Results

**Figure 3:**
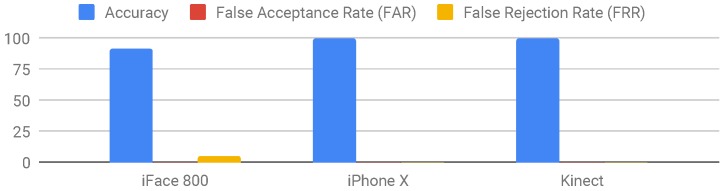
Overall results from the test-cases for the investigated 3D-camera devices.

After the user enrols her face as a new user and logs into the systems of the three devices, normally without spoof attacks or any alterations in appearance that might affect the process, the spoof attacks are presented and the results are reported. Table 5 and Figure 3 summarise the results.

In iFace 800, all spoof attacks were unsuccessful with an accuracy rate of 92%. Image size did not affect the detection process but distance, on the other hand, affected the process; if the distance between the image and the device is more than 50 cm then the device will not scan the face. This highlights how secure the device was against fraudulent attacks. However, the device was too rigid where any insignificant alteration in the appearance will affect the recognition process. The user cannot wear a scarf, makeup, glasses or change any minor thing in their appearance. By doing any of the above, the device will not recognise the user and will not check him/her in.

For iPhone X, we used the face recognition feature to unlock the phone. The attacks failed and the device succeeded in detecting all spoof attacks with an accuracy of 100%. Distance and size had an impact on the recognition process, if the user or fake face is more than 50 cm away from the camera (which is the maximum depth range), the scanning and detection process will terminate. The same action was applied to the size, if the image size was large (A4) the device stopped scanning for a face to detect. iPhone X face recognition process was not influenced by appearance alterations. The system recognised the user with or without scarf, with or without glasses and with or without makeup. The system was both secure and flexible, however, it only allows one user per device to register their face.

For the Kinect device, we implemented a system that utilises 3D sensor for depth information, the system was able to detect spoof attacks and deny access to the fake images. The size of the fake image had no influence on the spoof detection process, the system recognised the image as fake and did not allow it to log in. Additionally, alterations in the appearance does not affect the system. Wearing glasses, scarf, and makeup are all minor alterations and the user can log in. Our system with the Kinect sensor achieved 100%, which proves its reliability and flexibility. Kinect sensor has an extended depth range of 0.7 to 6 m. However, experiments of over 1 m distance test-cases show a decrease in anti-spoofing accuracy. Since the proposed software does not restrain authentication process based on face distance, the 3D face will be viewed as a flat plane with large distance.

Through our test cases, we concluded that iFace 800 was too strict in recognising genuine faces. Flexibility is important in such systems, the user should be able to freely change their appearance; wear eye lenses, hats, scarves, glasses and makeup without influencing the recognition process. However, it was important to take into consideration that if the flexibility is increased this might have a negative effect on the FAR. For this reason, a flexible face recognition system had to have robust security measurements. Furthermore, even though iPhone X and Kinect device were able to detect spoof attacks, distance restriction reduces the flexibility and the scalability of these systems.

## 6. Improvement Suggestions for Real Depth Liveness Authentication

In this research, our main aim was to investigate using real depth data as a liveness assurance technique. Using 3D cameras is effective in detecting spoof attacks and identifying faces, nevertheless, adding more constraints can improve the security measurements. Depth range in 3D stereo cameras (investigated in this research) depends on the distance between the device cameras and their resolution. Since distance is an important factor that influences the spoof detection process; where the system could not recognise the spoof attack, more research is needed to investigate how we can improve real depth liveness detection with distant faces. Even though a distance constraint, where the system scans faces within 25 to 50 cm away from the device, can limit the unrecognised attacks, it reduces the scalability of these systems. Research should investigate a solution where the system can identify and recognise spoof attacks regardless of the distance.

Robust facial recognition systems are recently being used in airports [47,48] and will be used in major athletic events [49], where visitors scan their face at the entry gate to access the country and access events. We can increase the scalability of real depth liveness detection technique and implement them in surveillance cameras. There are proposed techniques and researches of using face detection in video recordings. Developers have proposed an application for face recognition in videos, CascadeClassifier, FaceRecognizer and Fisherfaces method are used in their application for face recognition. However, this has not been tested against spoof attacks. If proved to be effective it can be merged with real depth liveness detection technique to measure its validity.

In [50], the authors proposed a face recognition system for videos. Their system is able to recognise faces regardless of the distance, which is a feature that is not fully utilised in real depth face detection systems. In their proposed system, videos are converted into frames and both successive mean quantisation transform (SMQT) feature and sparse network of winnows (SNoW) classifier are applied for face detection. For face recognition, the template matching technique is applied or what is sometimes referred to as linear spatial filtering. The FRR rate for their proposed system is high 12% and their proposed system does not use real depth data to detect spoof attacks. However, it is worth noting that distance did not affect the recognition process. The algorithm used in their paper can be useful and merged with real depth liveness assurance systems to overcome the distance barrier.

Moreover, in [51], the authors stated that high resolution images can be used to detect features of distant faces in crowded environments such as airports, stadiums and train stations. The authors presented a parallel stream-based implementation of Haar algorithm to detect faces in video sequences for identification. Their proposed method targets NVIDIA GPU, where videos are decoded and exposed to allow interoperability between the hardware-decoded video frames and CUDA kernel computations. The integral image is decoded and computed in parallel and evaluated. The coordinates of detected faces are delivered to the CPU for further processing and these results are bypassed to the conventional OpenGL pipeline using a pixel buffer object to display the results. The results of their work show that their parallel face detection framework performs better than existing work and achieves a frame rate of 35 fps at a resolution of 1920 × 1080 while decoding H.264 video in real-time. This method could possibly be implemented in real depth liveness assurance techniques, where a higher resolution of the faces would be taken and more frames per second would be analysed, strengthening the recognition process for faces that are more than 50 cm away from the camera.

## 7. Conclusions

Devices that depend on biometrics as an authentication method are evolving and being embedded more and more in individuals’ lifestyles. Facial features are used as an ID by even personal smart-phones and computers. This is one of many indications that the system is strongly secure. Nevertheless, attacks are effortlessly implemented in an attempt for unauthorised users to try to access these devices. Aforementioned attacks can be avoided using fake biometrics detection techniques, which are in constant growth and improvement. Using 3D cameras is one major anti-spoofing approach, where the system utilises 3D cameras to calculate the depth of the scanned face and determine its authenticity. Although in the research area, this approach is not extensively explored.

The aim of the work is to review existing 3D anti-spoofing techniques and highlight the strengths and weaknesses of 3D liveness detection technique for face recognition. The 3D anti-spoofing techniques were reviewed, where research that investigated existing techniques and implemented new suggestions was listed. SVM classifier, image quality assessment, raw data sensors, fisher score, pupil tracking and colour texture analysis all proved to be effective in identifying and preventing spoof attacks. The found anti-spoofing techniques are compared with one another in regards of datasets, used technique and results. Several projects investigated the effectiveness of 3D estimations in detecting liveness in face authentication, where the results were promising. However, none of these methods utilised real depth measurements in detecting fraud attacks.

Therefore, this research also explored face fraudulent detection using 3D cameras. This is done through conducting a proof-of-concept test cases on face recognition devices that use 3D and TrueDepth cameras. Our aim is to exploit its vulnerabilities and suggest solutions such as flexibility scale to improve the security and performance of real depth liveness detection technique. The results indicated that the existing 3D face recognition systems are reliable and secure. However, their strictness level needs to be adjusted for minor appearance change, such as wearing glasses. Moreover, face distance from the 3D camera had an effect in not recognising a face or wrongfully identifying fake attacks, since the face is treated as 2D flat image. Depth range of the 3D cameras is an important factor for bigger authentication/surveillance systems (e.g., airport, events). Selecting a 3D camera with high depth range and a high resolution can be a factor in enhancing face recognition process from the distant. This research area can be further investigated by creating a benchmark dataset that includes depth information beside the flat 2D images.

## Figures and Tables

**Table 1:** List of face authentication public datasets.

Dataset Name	Dataset Size	Dataset File	Spoofing Cases	Liveness Features
ZJU Eyeblink	80 video clips of 20 candidates	Video clips	4 clips per subject: a frontal view without glasses, a frontal view and wearing thin rim glasses, a frontal view and black frame glasses, and with upward view without glasses	Eyes blinking
Print-Attack Replay	200 video clips of 50 clients	Video clips, 2D images	Video attacks of clients, 2D printed-photo attack of clients	Real-access attempt videos
SLIET	65 video clips	Video clips, 2D images	videos of authentic users, pictures of valid users held by attacker	Eye blink, lips and chin movement
Replay-Attack Database	1300 photo and video attack attempts of 50 clients	Video clips, 2D images	Video attacks of clients under different lighting, 2D printed-photo attack of clients under different lighting, On-screen attacks	25 general image quality measures and Context-based using correlation between face motion vs. background motion
NUAA	12,614 of live and photographed faces	2D images	2D printed photos	Face texture using Lambertian model and Eye blink detection using Conditional Random Fields
CASIA	50 subjects	video recordings of genuine and fake faces	warped photo attacks, cut photo attacks, video attacks	Facial motion and eyes blinking
3DMAD	76,500 frames of 17 persons	depth image, RGB image	Video attacks, 3D mask attacks	Eye position
Patrick Chan	50 subjects 600 videos	2D images	paper photo attack, iPad photo attack, video attack, 2D mask attack, curved mask attack	Temperature
MSU MFSD	280 video clips of photo and video attack attempts to 35 clients	Video clips	printed attacks, replay attacks generated with a mobile phone and a tablet	Image distortion based quality measures
Replay-mobile database	1030 videos from 40 subjects	Video clips	print photo attack, tablet photo attack, mobile video attack	Image resolution
IIIT-D	4603 RGB-D images of 106 subjects	RGB Depth Images	Flat images	Pose and expression variation
BUAA Lock3DFace	5711 RGB-D video sequences of 509 subjects	RGB Depth Videos	Video attacks	Face2D liveness detection
Jiyun Cui	RGB-D images of 747 subjects	RGB Depth Images	Flat images	2D face recognition
SMAD	130 video	Video clips	vivid silicone masks	Dynamic Texture for 3D Mask
Oulu-NPU	4950 bona fide and artefact face videos corresponding to the 55 subjects	Video clips, 2D images	Print photo, Replay video	Image resolution
Yale-Recaptured	28 subjects	Images	LCD screen	Different resolutions
MS-Face	21 subject	Images	Printed images	Colour
Florence 2D/3D Face	53 subject	3D scans of human faces Videos and images	No spoof attack	N/A

**Table 2:** Related work summary.

Ref	Biometrics	Spoof Attack	Anti-Spoofing	
			Hardware	Software
[18]	Face biometrics	Shaking a regular photograph in front of the camera and real time video on a mobile phone	Basic hardware equipment	Pupil Tracking
[10]	Iris, Fingerprint, Face Recognition	Synthetic iris samples, A database of fake fingerprint, A database containing short videos of face images	-	Image quality assessment
[9]	Face biometrics	3D mask, print and video	Pulse detection from facial videos	Analyse colour changes corresponding to heart rate
[12]	Face biometrics	Two cameras: one that captures an infrared, image and another which captures a normal image	Using light reflection—infrared led	-
[19]	Face biometrics	Images presented on smart-phone screen	-	Raw data sensors
[20]	Iris, Face, Palm images	Fusion Technique	-	Image quality assessment
[21]	Fingerprint, Face	Fake fevicol fingerprint, Mobile Images both high and low resolution, Photo print image	-	Image quality assessment
[22]	Face biometrics	A regular live video, printed pictures, wearing a mask with printed pictures, pictures in a LCD monitor, video displayed in mobile devices	-	Similarity is measured (using similarity index measure (SSIM) and a background motion index (BMI))
[23]	Face biometrics	Genuine photo, planar photo, photo wrapped horizontally and photo wrapped vertically	-	Analysing 3D structure
[24]	Face biometrics	Face images printed on A4 paper	-	Non-rigid motion, face-background consistency, banding effect.
[25]	Face biometrics	Imitating high resolution photographs in motion	-	Utilising optical flow of lines
[26]	Face biometrics	Fake face images are taken from faces printed on paper or displayed on an LCD screen	-	Using noise local variance model
[27]	Face biometrics	2D printed photograph presented either wrapped or unwrapped	-	Image quality assessment
[28]	Face biometrics	Wrapped photo attack, cut photo attack, video attack	-	Spectral analysis
[29]	Face biometrics	Print and replay attacks from live (valid)videos containing an authentic face	-	Dynamic Mode Decomposition
[30]	Iris Face Fingerprint	Printed images	-	Using hyper-parameter optimisation of network architectures ( architecture optimisation)
[31]	Face biometrics	Used spoofing attacks provided in these databases: CASIA, Replay-Attack, NUAA	-	Using Multiscale Dynamic Binarized Statistical Image Features
[32]	Face biometrics	Printed faces, video displays or masks	-	Colour texture analysis
[33]	Face biometrics	A set of polarised images, And printed paper masks	Polarised light	
[34]	Face biometrics	Image displayed on iPad, image displayed on iPhone, and printed photo	-	Image distortion analysis
[35]	Face biometrics	Using the Extended Multispectral Presentation Attack Face Database (EMSPAD)	-	analysing the spectral signature
[36]	Face biometrics	Warped photo attack, cut photo attack and video attack	-	Fisher score
[37]	Face biometrics	2D photographs, video attacks and masks	-	HOG-LPQ and Fuz-SVM Classifier
[38]	Face Biometrics	3D Printed Masks		ArtecID 3D Face Log On System
[39]	Face Biometrics	A4 paper photo attack, iPad photo attack, video attack, mask attack, and curved mask attack	Flash light	Light illumination detection
[40]	Face Biometrics	video and photo attacks	-	Eyeblink, lip movement and chin movement, background changes
[41]	Face Biometrics	Different environments and lighting conditions	Flash light	Computing 3D features
[42]	Face Biometrics	video attacks	-	Deep CNN networks
[14]	Face Biometrics	2D images	-	Depth estimation
[43]	Face Biometrics	2D images, video clips attacks	-	Guided scale space to reduce noise
[16]	Face Biometrics	video attacks	-	Colour texture analysis
[15]	Face Biometrics	3D mask attacks	-	Deep dynamic texture
[44]	Face Biometrics	2D images, video clips attacks	-	Nonlinear diffusion
[45]	Face Biometrics	2D images, iPad image display attacks	-	Characteristics of the display and probabilistic neural network

**Table 3:** Summary of spoof attacks.

Spoof Attack	Material
Real face	Real face wearing scarf (Baseline)
	Real face without scarf
	Real face wearing glasses (Occlusion)
Face displayed on device	iPhone 6s Screen Huawei Tablet Lenovo Laptop
Face printed on A4 image paper	Matte Glossy
Printed Face mask (bent and worn on the face)	Matte Glossy
Video attack - face moving and eyes blinking	iPhone 6s Screen Huawei Tablet Lenovo Laptop

**Table 4:** Device screen specifications.

	iPhone 6s	Huawei Tablet	Lenovo Laptop
Type	LED-backlit IPS LCD Capacitive touchscreen 16 M colors	IPS LCD Capacitive touchscreen 16 M colors	HD LED TN Glare Flat eDP
Size	4.7 inches	7.0 inches	14 inch
Resolution	750 × 1334 pixels	600 × 1024 pixels	1366 × 768 pixels

**Table 5:** Summary of Spoof Attack Results.

Spoof Attack	Material	Ground Truth *	Results		
			iFace 800	iPhone X	Kinect
Real Face	Real face with scarf	Accept	Accept	Accept	Accept
	Real face wearing glasses	Accept	Reject	Accept	Accept
	Real face without scarf	Accept	Reject	Accept	Accept
Face displayed on device	iPhone 6s screen	Reject	Reject	Reject	Reject
	Huawei tablet	Reject	Reject	Reject	Reject
	Lenovo laptop	Reject	Reject	Reject	Reject
Printed Face image	Matte	Reject	Reject	Reject	Reject
	Glossy	Reject	Reject	Reject	Reject
Printed Face mask(bent and worn on face)	Matte	Reject	Reject	Reject	Reject
	Glossy	Reject	Reject	Reject	Reject
Video attack	iPhone 6s screen	Reject	Reject	Reject	Reject
	Huawei tablet	Reject	Reject	Reject	Reject
	Lenovo laptop	Reject	Reject	Reject	Reject

* Accept: indicates successfully given access; Reject: indicates failure to give access.
